# Supplementary material for: Validation of a Dendritic Cell and CD4+ T Cell Restimulation Assay Contributing to the Immunogenicity Risk Evaluation of Biotherapeutics
Source: Pharmaceutics. 2022 Dec 1;14(12):2672. doi: 10.3390/pharmaceutics14122672 (PMC9781343; doi:10.3390/pharmaceutics14122672)
Supplement: Supplementary file 1 [file pharmaceutics-14-02672-s001.zip › pharmaceutics-2034956-SI.pdf]

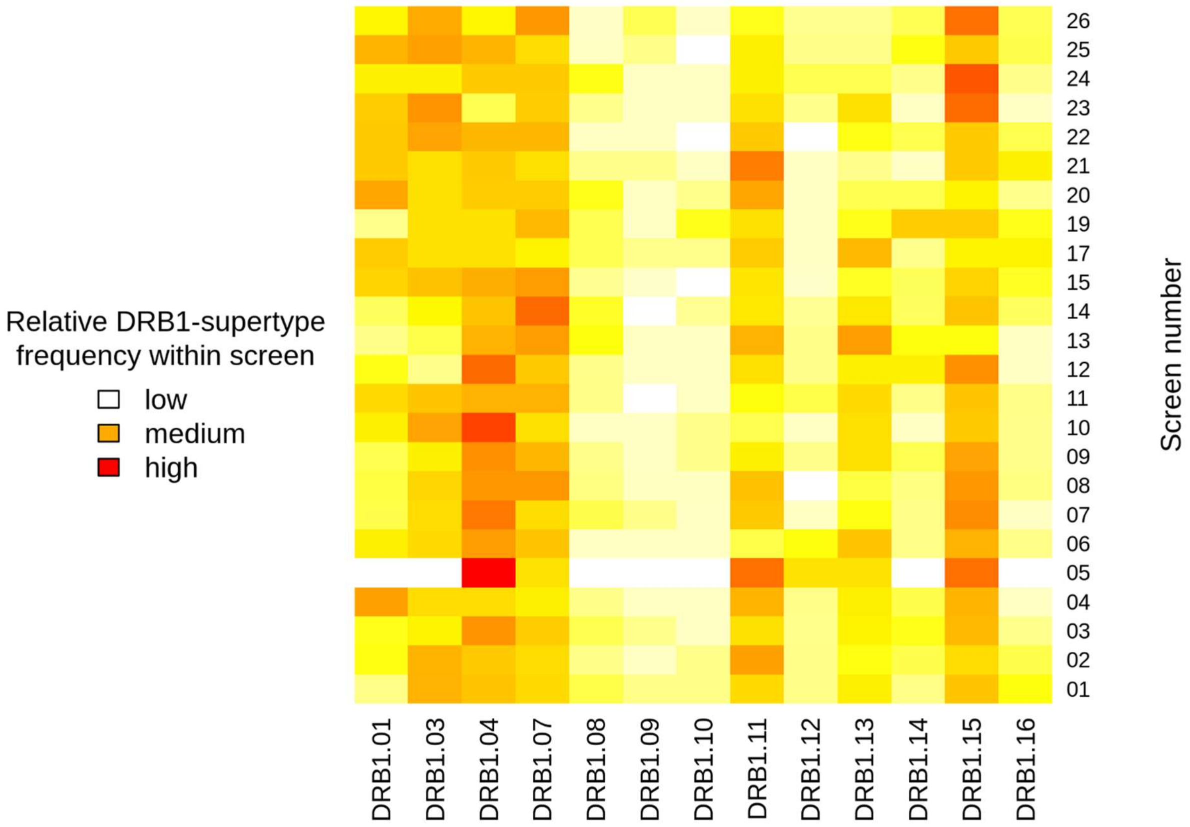


**Figure S1. Heatmap showing the relative distribution of the HLA-DRB1 supertype frequencies among the donor cohorts per screen of the DC:CD4+ T cell restimulation assay.** Each screen is represented by a row, and each HLA-DRB1 supertype by a column. For each screen, the relative HLA-DRB1 supertype frequencies were calculated to allow comparison across screens with different cohort sizes. The darker the color in the heatmap, the higher the relative frequency of the respective HLA-DRB1 supertype within a screen. For instance, HLA-DRB1:04 and HLA-DRB1:15 typically appeared frequently compared to other HLA-DRB1 supertypes in the cohorts of examined screens, whereas HLA-DRB1:09 and HLA-DRB1:10 appeared relatively rarely. Consequently, this analysis shows that – despite the fact that typically different donors with different HLA-DRB1 supertypes form the cohorts of each screen – a relatively similar distribution of HLA-DRB1 supertype frequencies is achieved at the cohort level across screens. This relative distribution is selected to follow that of the world population [5].
